# Temporal trends in acute coronary syndrome among women and association with socioeconomic factors—evidence from a middle-income country

**DOI:** 10.3389/fgwh.2026.1750182

**Published:** 2026-06-02

**Authors:** Roberta Marković, Aleksandra Ignjatović, Ivan Vučić, Aleksandar Višnjić, Tamara Jovanović, Marija Andjelković Apostolović, Sonja Dakić, Suzana Otašević

**Affiliations:** 1 Department of Social Medicine, Faculty of Medicine, University of Niš, Niš, Serbia; 2 Institute of Public Health of Niš, Niš, Serbia; 3 Department of Medical Statistics, and Informatics, Faculty of Medicine, University of Niš, Niš, Serbia; 4 Institute of Public Health of Leskovac, Leskovac, Serbia; 5 Department of Internal Medicine and Health Care, Faculty of Medicine, University of Niš, Niš, Serbia; 6 Department of Microbiology, Faculty of Medicine, University of Niš, Niš, Serbia

**Keywords:** women, acute coronary syndrome, socioeconomic factors, middle-income country, temporal trends

## Abstract

**Introduction:**

Since Acute Coronary Syndrome (ACS) death rates remain a challenge underscoring the importance of socioeconomic factors, the aim of the study was to explore the trend in incidence, mortality, and mortality-to-incidence ratio (MIR) of ACS and Myocardial Infarction (MI) among women in Serbia, middle income country, from 2006 to 2022, as well possible association with the Human Development Index (HDI), Social Demographic Index (SDI), and Years of Life Lost (YLL).

**Methodology:**

The research was conducted according to the principles of a descriptive epidemiological study, using data extracted from publicly accessible yearbooks, registry and reports (count, and age-standardized rates). Statistical analysis was performed using Joinpoint Regression analysis with the Joinpoint Regression Program version 5.4.

**Results and discussion:**

There were a significant declining trend of MI incidence (APC −2.1, *p* = 0.005) and mortality rates (APC −7.8, *p* < 0.001); ACS incidence did not change significantly, while ACS mortality decreased (APC −6.8, *p* < 0.05). There was significant association between trend of ACS and MI incidence, mortality and MIR at women in Serbia, and increasing trend of HDI and SDI. The constant decline in YLL followed, but the number of lost years remains high (APC −5.9, *p* < 0.001).

**Conclusion:**

Consistently high mortality rates from ACS and MI among women in Serbia may be attributed to the complex phases of socioeconomic transformation the country has experienced, characterised by high exposure to risk factors and insufficient health promotion and prevention strategies. Urgently prioritising cost-effective, multidisciplinary prevention strategies for women, adapted to local contexts and aligned with health and other Sustainable Development Goals, is critical to reducing global disparities in cardiovascular outcomes.

## 1 Introduction

Cardiovascular diseases (CVD) were responsible for 20.5 million deaths in 2021. year, nearly one-third of all deaths across the globe, which is alarming considering that up to 80% of premature heart attacks and strokes can be prevented (1). The number of people living with CVD almost doubled between 1990 and 2019, increasing from 271 million to 523 million (1), although the global age-standardized prevalence rate of cardiovascular diseases (CVD) declined overall. However, trends varied by disease type, socio-demographic development, and age group (2). While CVD increases were observed in rheumatic heart disease, non-rheumatic valvular heart disease, hypertensive heart disease, and other cardiovascular conditions, the overall burden decreased significantly in high-middle and high SDI countries and increased in middle, low-middle, and low SDI countries (3). Age-specific patterns showed declining prevalence rates among older populations (50–69 and ≥70 years) but increasing rates among younger groups (0–14 and 15–49 years) (4). These findings highlight substantial disparities in CVD burden across regions and age groups.

One of the major groups of CVDs, acute coronary syndromes (ACS), as life-threatening conditions that embrace unstable angina and myocardial infarction (MI), has been seen as a disease of primary health concern in the general population throughout the past several decades (5). Although ACS death rates have decreased in numerous developed countries over the past decades, they remain a challenge in low- and middle-income countries, underscoring the importance of socioeconomic factors in cardiovascular health (2, 6). In the Republic of Serbia, an upper-middle-income country in Southeast Europe (7), for years, CVDs have been responsible for half of deaths; in 2021, it was ranked first in overall mortality, causing 51.624 (47.3%) deaths. Among those were 18.3% died of ischemic heart disease (IHD), within which 48.3% people died of acute coronary syndrome (ACS), which corresponds to an age-standardised mortality rate (ASR) of 736.9/100,000 for females, and 873.5/100,000 for males (8). According to the European Society of Cardiology, Serbia is considered a “very high-risk” country with ≥300 CVD deaths per 100,000 population (9)*.*

Extensive research has been carried out on CVD and ACS risk factors in low, middle and high income countries, and has documented common modifiable risk factors such as tobacco smoking, sedentary lifestyle, hyperlipidemia, hypertension, diabetes, obesity, as well as non-modifiable risk factors such as age and gender (10). Many research papers underlined gender-based disparities in risk exposure, ACS treatment and outcomes (11, 12), and determined ACS in women as a significant public health concern worldwide (13, 14). Women are a vulnerable group for ACS due to a combination of biological factors, pathophysiology of ACS in women, risk awareness, disease presentation, medical treatment and influence of social factors (5, 15, 16). However, despite similar risk factors, CVD outcomes occur within the context of a country's socioeconomic characteristics (10), which has been analyzed in previous studies (2, 17–19), but a better understanding of this relationship is recognized as a key for assessing existing weaknesses and developing future directions for preventive strategies related to health (6, 10).

Some socioeconomic factors are comprised within the Human Development Index (HDI) and the Social Demographic Index (SDI). The United Nations Development Program's (UNDP) HDI, which associates a country's per-capita gross national income (GNI), mean life expectancy of the population, and mean or expected years of schooling of the country's citizens, remains a standardized measure of the developmental status of an individual country (20). The HDI was introduced to quantify the development level within regions and to identify social inequalities between countries more precisely. SDI gathers data on the economy, education, and fertility rates of countries worldwide and emphasizes information on the compound interaction between socio-economic factors and health. At the beginning of the nineties, HDI had a downward trend in Serbia, and it was classified as a country with medium HDI values. Between 1995 and 2022, Serbia's HDI value increased from 0.675 to 0.805, representing a 19.3 per cent change (20). According to the UNDP report, Serbia's HDI in 2022. was 0.805, which places it in 65th place on the list of 193 countries and territories included in the report, and the category of very highly developed countries. The level of SDI in 2019 classifies Serbia in the group with middle-high SDI (21).

Since the prevention of premature mortality is considered as a public health objective, additional analyses regarding years of life lost (YLL) due to ACS mortality in women are required to support the development of health policies. YLL is a standard indicator often used to measure a component of premature mortality that can be avoided. Likewise, YLL provides useful information to explore its possible association with socioeconomic factors influencing health (22).

There are a few studies related to mortality from CVD and ACS in some Serbian districts, as well as temporal trends in ACS mortality (23–25), but there is scarce up-to-date evidence and understanding on the trend of the relationship between socioeconomic factors and ACS in women, over a specific period. Hence, the aim of this study was to examine trends in the incidence, mortality, and mortality-to-incidence ratio (MIR) of acute coronary syndrome (ACS) and myocardial infarction (MI) among women in Serbia during the period 2006–2022. In addition, the study sought to investigate the potential association between these rates and socioeconomic indicators, perceived through the Human Development Index (HDI), the Socio-demographic Index (SDI), and years of life lost (YLL) attributable to these conditions. Understanding these relationships may contribute to a more comprehensive assessment of the burden of ACS and MI among women and may help identify globally relevant, targeted public health strategies and cost-effective policies aimed at improving prevention, early detection, and management of ACS in the female population.

## 2 Methods

### 2.1 Study area

The Republic of Serbia is situated in Southeast Europe, covers a territory of 88,499 km^2^, with 6,567,783 citizens (51.4% female and 48.6% male), according to the Statistical Office of the Republic of Serbia (https://data.stat.gov.rs/?caller=SDDB&languageCode=en-US). The population of Serbia is characterized by a negative demographic trend (a decline in the population by 12% over the period 2002–2024), reflected in a negative population growth rate (−5.7/1,000), a low fertility rate (1.63), and a pronounced aging process (average age 44.0). High share of people aged 65 and over is continuously increasing (from 17,4% (2011) to 22,1% (2022), indicating significant demographic aging and places the country among the oldest in Europe. Life expectancy at birth, according to abridged life tables year is 73,70 for male and 78,35 for female (below the average of European Union countries). Beside the challenge of an ageing population, Serbia's community has been facing dominant burden of noncommunicable diseases (malignant tumors, diabetes, chronic obstructive pulmonary disease, injuries), and cardiovascular diseases as the primary cause of death nationwide (Health Statistical Yearbook of Republic of Serbia, 2024., available at https://www.batut.org.rs/download/publikacije/pub2024v2.pdf).

### 2.2 Study design and data collection

The research was conducted according to the principles of a descriptive epidemiological study, using data extracted from publicly accessible Health Statistical Yearbook of Republic of Serbia from the Institute for Public Health of Serbia, ˝Dr Milan Jovanovic Batut˝, for the period 2006–2022, (available at https://www.batut.org.rs/index.php?content=77), and the Incidence and mortality of Acute Coronary Syndrome in Serbia from the Institute for Public Health of Serbia, ˝Dr Milan Jovanovic Batut˝, for the period 2006–2022. (available at https://www.batut.org.rs/index.php?category_id=9). ACS and MI are coded according to the 10th Revision of the International Classification of Diseases (ICD−10).

Two sociodemographic indexes were extracted: the HDI for Serbia in the period 2006–2022 from the United Nations Human Development Reports (20), and the SDI for Serbia in the period 2006–2022 from the Global Health Data Exchange, Institute for Health Metrics and Evaluation (21). YLL was calculated using data from the publications, “The Incidence and Mortality of Acute Coronary Syndrome in Serbia” (2006–2022). YLL was calculated by multiplying the number of deaths in each age group by the standard life expectancy at the corresponding age, according to the Global Burden of Disease methodology.

The study did not involve a predefined sample size calculation, as all available cases recorded in the national registry were included. In the Incidence and mortality of Acute Coronary Syndrome in Serbia registry, it is specified that the diagnosis of ACS is established, followed by the recommendations of the European Society of Cardiology (26, 27).

### 2.3 Statistical analyses

Data are presented as counts and percentages. Crude rates and age-standardized rates (ASR-W) were extracted from yearbooks. Statistical analysis of the trend was performed using joinpoint regression analysis to calculate the trend, the annual percentage change (APC) of incidence, mortality, and YLL. In joinpoint regression analysis, the optimal number of joinpoints was estimated using a Monte Carlo permutation method. The trend was considered significant when the *p*-value was less than 0.05. Possible associations between the socioeconomic indicators HDI and SDI and age-standardised rates of incidence, mortality, and MIR were assessed using simple regression analysis. Collinearity diagnostics were not performed because each regression model included only one showed independent variable. The methodological approach in analysing trends and estimating potential association between incidence and mortality rates with socioeconomic indicators has been commonly used in previous research (28–31). The YLL values were determined by multiplying the mortality figures for each age group by the corresponding life expectancy and then summing the results. Joinpoint Regression analysis was performed with the Joinpoint Regression Program version 5.4.0 (available at http://surveillance.cancer.gov/joinpoint). Simple regression analysis and graphical data presentation was performed using the statistical software R (v. 4.3.0) (32).

## 3 Results

Supplementary Tables S1, S2 show the number of women with MI and ACS in specific age categories, i.e., the number of deceased women with MI and ACS by age category. Table 1 shows crude rates and ASR-W incidence rates for MI and ACS for women of different ages.

**Table 1:** Joinpoint regression analysis—MI and ACS MIR among women in Serbia in 2006–2022.

	Group	Lower Endpoint	APC	95% CI	*p*	AAPC	95% CI	*p*
ASR-W 25–64	MI	2006–2010	−10.0	−21.4–3.1	0.118	−5.3*	−8.5 to −1.9	0.002
	MI	2010–2022	−3.7*	−6.1 to −1.2	0.008			
	ACS	2006–2011	−7.5*	−14.2 to −0.2	0.044	−5.2*	−7.6 to −2.8	0.001
	ACS	2011–2022	−4.2*	−6.3 to −2.0	0.002			
ASR-W 0–64	MI	2006–2010	−9.8	−21.3–3.4	0.125	−5.2*	−8.5 to −1.9	0.003
	MI	2010–2022	−3.7*	−6.1 to −1.2	0.008			
	ACS	2006–2011	−7.6*	−14.2 to −0.5	0.038	−5.2*	−7.5 to −2.8	0.001
	ACS	2011–2022	−4.1*	−6.2 to −1.9	0.002			
ASR-W 0–75+	MI	2006–2010	−8.3	−16.3–0.6	0.064	−4.0*	−6.3 to −1.7	0.001
	MI	2010–2022	−2.6*	−4.2 to −0.9	0.006			
	ACS	2006–2008	7.7	−23.8–52.3	0.648	−2.4	−6.3–1.7	0.246
	ACS	2008–2022	−3.8*	−5.3 to −2.2	0.001			

ASR-W, age standardized rate; APC, annual percent change; AAPC, average annual percent change; 95% CI, 95% confidence interval; * significant change.

Joinpoint regression analysis showed that there was a statistically significant decrease in the trend of MI incidence until 2017 (ASR W 25–64, ASR W 0–64, ASR W 0–75). The incidence of ACS does not change statistically significantly during the follow-up period (Table 2). Joinpoint regression analysis showed that there is a statistically significant decrease in the mortality trend from MI until 2019 (ASR W 25–64), until 2011 (ASR W 0–64), and, until 2016 (ASR W 0–75). ACS mortality is statistically significantly reduced in the period 2006–2011 (ASR W 25–64, ASR W 0–75), i.e., in the entire period (ASR W 0–64) (Table 2), i.e., 2016 (ASR W 0–75).

**Table 2:** Joinpoint regression analysis—MI and ACS INCIDENCE and mortality among women in Serbia in 2006–2022.

	Incidence											
	**ASR-W 25-64**				**ASR-W 0-64**				**ASR-W 0-75**			
	**Analyzed Period**	**APC**	**95% CI**	**p**	**Analyzed Period**	**APC**	**95% CI**	**p**	**Analyzed Period**	**APC**	**95% CI**	**p**
MI	2006-2017	−1.9*	−3.4—0.3	0.024	2006–2017	−2.3*	−3.9– −0.7	0.012	2006–2017	−2.1*	−3.4– −0.8	0.005
	2017-2022	2.3	−2.9–7.8	0.361	2017–2020	7.1	−16.3–37.0	0.545	2017–2022	1.3	−3.1–5.9	0.537
					2020–2022	−7.6	−27.8–18.2	0.487				
ACS	2006-2010	26.6	−12.3–82.9	0.180	2006–2010	23.3	−9.7–68.4	0.169	2009–2013	−4.20	−10.8–2.8	0.203
	2010–2013	−5.1	−70.3–203.5	0.921	2010–2022	−0.1	−5.8–5.9	0.968	2013–2022	0.40	−1.7–2.4	0.691
	2013–2022	1.2	−9.0–12.3	0.800								

	Mortality											
	ASR-W 25-64				ASR-W 0-64				ASR-W 0-75			
	Analyzed Period	APC	95% CI	p	Analyzed Period	APC	95% CI	p	Analyzed Period	APC	95% CI	p
MI	2006-2011	−10.6*	−15.6– −5.2	0.002	2006–2011	−10.9*	−20.4– −6.7	<0.001	2006–2011	−7.8*	−10.1 – −5.4	<0.001
	2011-2019	−4.2*	−7.5– −0.8	0.020	2011–2022	−3.9	−5.2 −0.7	ns	2011–2016	−5.9*	−9.2 – −3.8	0.004
	2019-2022	−2.0	13.9–11.5	0.728					2016–2022	−0.9	−2.8 – 1.0	0.292
ACs	2006-2011	−10.6*	−20.9 – −6.2	<0.05	2006–2022	−5.6*	−6.7 – −10.9	<0.001	2006–2016	−6.8*	−7.9– −6.0	<0.05
	2011-2022	−4.0	−5.4 – 2.2	Ns					2016–2022	−0.5	−2.2–2.5	ns

ASR-W, age standardized rate; AAPC, average annual percent change; 95% CI, 95% confidence interval; * significant change.

**Figure 1:**
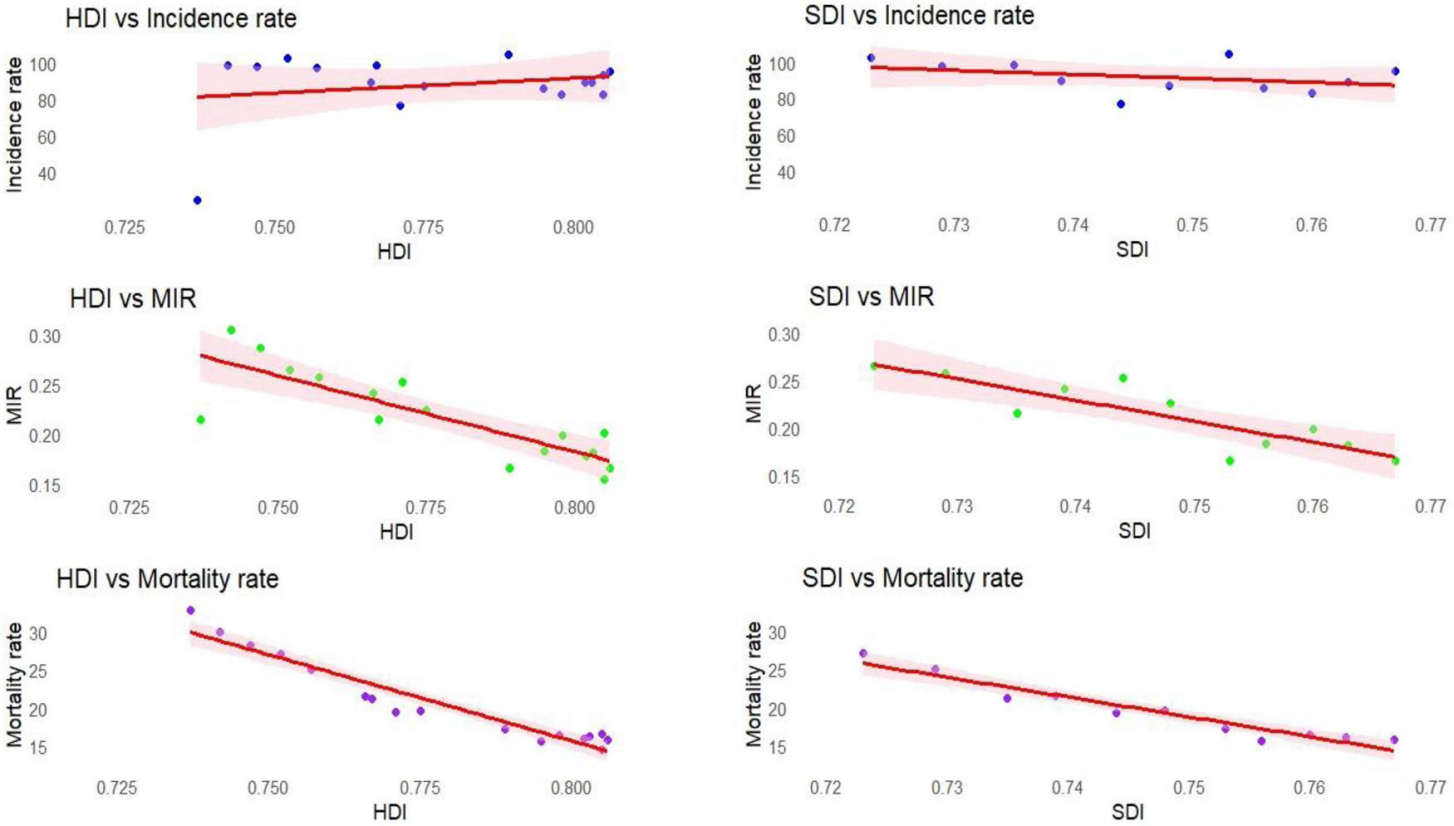
Association between HDI, SDI and ACS incidence rate, mortality rate and MIR among women in Serbia.

HDI was found to be statistically significantly associated with MI incidence (ASR W 0–64, ASR W 0–75+). HDI is statistically significantly associated with MIR for IM (ASR W 25–64, ASR W 0–64, ASR W 0–75+), and ACS (ASR W 25–64, ASR W 0–64, ASR W 0–75+). HDI is statistically significantly associated with mortality rates for IM (ASR W 25–64, ASR W 0–64, ASR W 0–75+), and ACS (ASR W 25–64, ASR W 0–64, ASR W 0–75+) (Table 3). SDI was found to be statistically significantly associated with the incidence of MI (ASR W 25–64, ASR W 0–64, ASR W 0–75+). SDI is statistically significantly associated with MIR for MI (ASR W 25–64, ASR W 0–64, ASR W 0–75+). SDI is statistically significantly associated with Mortality rates for IM (ASR W 25–64, ASR W 0–64, ASR W 0–75+), and ACS (ASR W 25–64, ASR W 0–64, ASR W 0–75+) (Table 4), (Figure 1).

**Table 3:** Association between HDI and incidence, mortality rates, and MIR.

	Model		Incidence					R^2^	MIR					R^2^	Mortality					R^2^
			B	Beta	95.0% CI for B		p	adjustedR^2^	B	Beta	95.0% CI for B		p	adjustedR^2^	B	Beta	95.0% CI for B		p	adjustedR^2^
MI	ASR-W 25–64	Constant	165.6		63.4	267.9	0.004	0.220	1.8		1.3	2.3	<0.001	0.778	144.0		116.9	171.0	<0.001	0.876
		HDI	−127.0	−0.5	−258.5	4.4	0.057	0.168	−2.1	−0.9	−2.7	−1.5	<0.001	0.763	−168.1	−0.9	−202.9	−133.4	<0.001	0.868
	ASR-W 0–64	Constant	83.0		32.9	133.1	0.003	0.235	1.8		1.3	2.3	<0.001	0.781	70.6		57.5	83.6	<0.001	0.880
		HDI	−64.9	−0.5	−129.3	−0.5	0.048	0.184	−2.1	−0.9	−2.7	−1.5	<0.001	0.766	−82.5	−0.9	−99.3	−65.7	<0.001	0.872
	ASR-W 0–75	Constant	207.8		116.8	298.8	<0.001	0.421	2.1		1.7	2.6	<0.001	0.821	190.6		162.4	218.7	<0.001	0.918
		HDI	−181.1	−0.6	−298.1	−64.1	0.005	0.382	−2.4	−0.9	−3.0	−1.8	<0.001	0.810	−219.4	−1.0	−255.5	−183.2	<0.001	0.912
ACS	ASR-W 25–64	Constant	−58.4		−351.0	234.2	0.677	0.075	1.3		1.1	1.6	<0.001	0.875	148.1		122.4	173.9	<0.001	0.892.
		HDI	194.2	0.3	−182.0	570.4	0.288	0.013	−1.5	−0.9	−1.8	−1.2	<0.001	0.867	−173.1	−0.9	−206.2	−139.9	<0.001	0.885
	ASR-W 0–64	Constant	−27.5		−170.6	115.7	0.688	0.072	1.3		1.1	1.6	<0.001	0.880	72.4		59.8	85.0	<0.001	0.892
		HDI	93.3	0.3	−90.7	277.3	0.297	0.010	−1.5	−0.9	−1.8	−1.2	<0.001	0.872	−84.6	−0.9	−100.8	−68.4	<0.001	0.885
	ASR-W 0–75	Constant	−37.8		−344.3	268.6	0.796	0.049	1.4		1.0	1.8	<0.001	0.705	197.3		169.8	224.8	<0.001	0.926
		HDI	161.9	0.2	−232.2	555.9	0.395	−0.015	−1.5	−0.8	−2.1	−1.0	<0.001	0.685	−227.0	−1.0	−262.3	−191.6	<0.001	0.921

ASR-W, age standardized rate; B, unstandardized regression coefficient; Beta, standardized regression coefficient; R^2^, adjusted ratio coefficient; 95% CI, 95% confidence interval.

**Table 4:** Association between SDI and incidence, mortality rates, and MIR.

	Model		Incidence					R^2^	MIR					R^2^	Mortality					R^2^
			B	Beta	95.0% CI for B		p	adjustedR^2^	B	Beta	95.0% CI for B	p		adjustedR^2^	B	Beta	95.0% CI for B		p	adjustedR^2^
MI	ASR-W25–64	Constant	217.5		94.8	340.2	0.002	0.374	1.9		1.3	2.6	<0.001	0.750	165.3		137.7	192.9	<0.001	0.922
		SDI	−203.9	−0.6	−369.9	−37.9	0.020	0.322	−2.3	−0.9	−3.2	−1.5	<0.001	0.729	−204.7	−1.0	−242.1	−167.4	<0.001	0.916
	ASR-W0–64	Constant	109.1		49.2	169.0	0.002	0.393	1.9		1.3	2.5	<0.001	0.749	80.8		67.4	94.2	<0.001	0.924
		SDI	−103.6	−0.6	−184.7	−22.6	0.016	0.342	−2.3	−0.9	−3.1	−1.5	<0.001	0.728	−100.1	−1.0	−118.2	−82.0	<0.001	0.917
	ASR-W0–75	Constant	238.9		117.3	360.4	0.001	0.440	2.4		1.8	3.0	<0.001	0.833	219.2		197.0	241.4	<0.001	0.969
		SDI	−231.6	−0.7	−396.1	−67.2	0.010	0.393	−2.8	−0.9	−3.6	−2.0	<0.001	0.819	−268.1	−1.0	−298.1	−238.0	<0.001	0.967
ACS	ASR-W25–64	Constant	−109.2		−517.0	298.6	0.570	0.088	1.4		1.1	1.7	<0.001	0.837	169.2		143.2	195.2	<0.001	0.933
		SDI	271.7	0.3	−280.0	823.4	0.304	0.012	−1.7	−0.9	−2.2	−1.2	<0.001	0.824	−209.4	−1.0	−244.6	−174.2	<0.001	0.928
	ASR-W0–64	Constant	−51.6		−251.2	147.9	0.583	0.084	1.4		1.1	1.7	<0.001	0.840	82.8		70.2	95.5	<0.001	0.934
		SDI	130.2	0.3	−139.8	400.2	0.314	0.008	−1.7	−0.9	−2.2	−1.2	<0.001	0.827	−102.6	−1.0	−119.7	−85.4	<0.001	0.929
	ASR-W0–75	Constant	−134.3		−558.1	289.6	0.503	0.098	1.4		0.8	2.1	<0.001	0.577	226.0		203.7	248.2	<0.001	0.971
		SDI	300.8	0.3	−272.7	874.2	0.275	0.023	−1.6	−0.8	−2.5	−0.7	0.002	0.542	−276.2	−1.0	−306.2	−246.1	<0.001	0.968

ASR-W, age standardized rate; B, unstandardized regression coefficient; Beta, standardized regression coefficient; R^2^ – adjusted ratio coefficient; 95% CI, 95% confidence interval.

**Figure 2:**
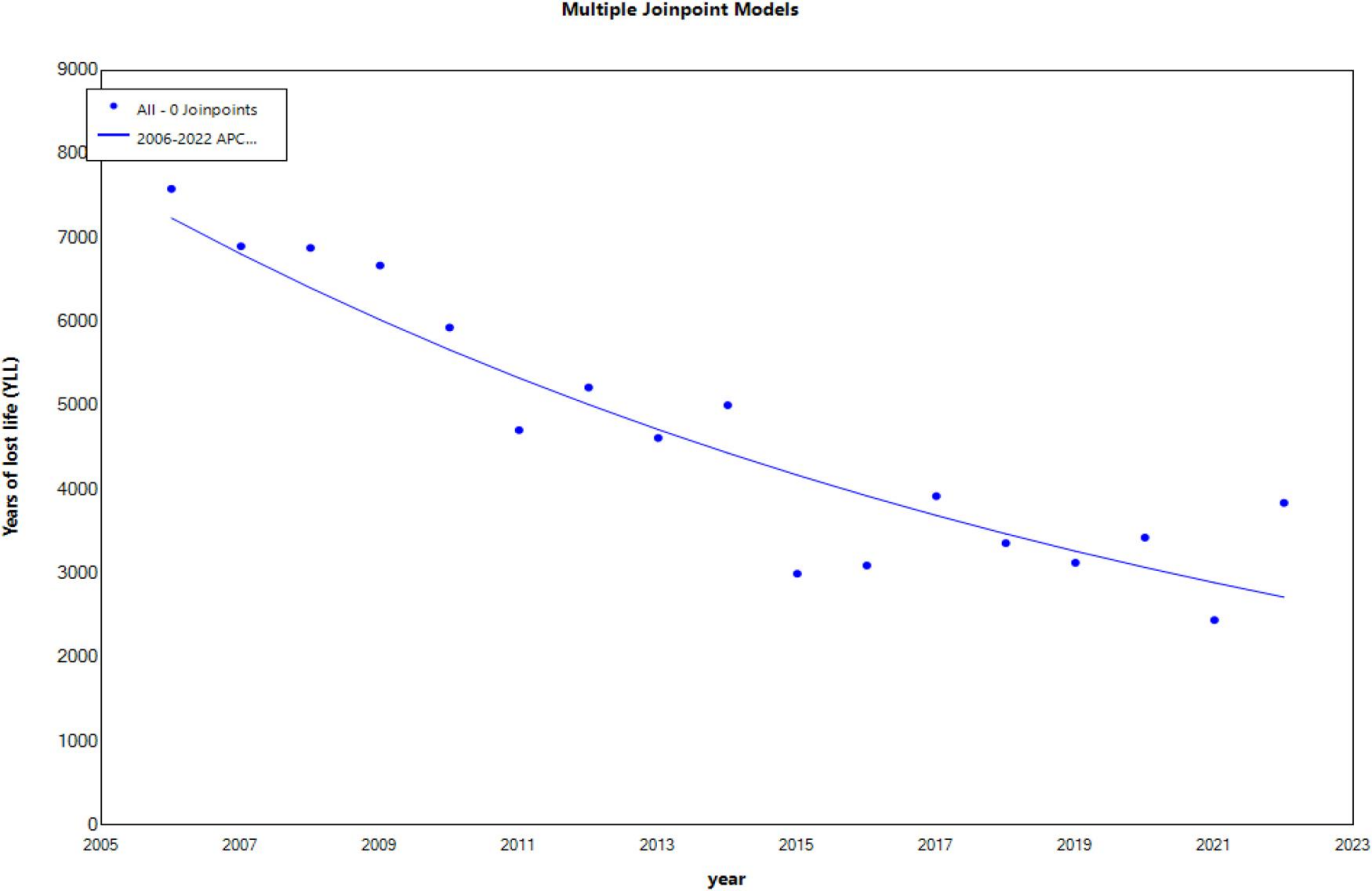
YLL trend of ACS among women in Serbia in 2006-2022.

The total YLL for the observed period is 79716.9, of which 42.6% was lost in the first five years of monitoring, and only 11.9% in the last five years of the observed period. In Supplementary Table S3 shows YLL values by year. It was found that there was a statistically significant decrease in YLL trend for ACS during the studied period, with an APC of −5.94 (95% CI −7.5 to −4.4, *p* < 0.001) (Figure 2).

## 4 Discussion

In recent times, analyses of the burden of cardiovascular diseases have gone beyond the analysis of just physiological aspects of the disease and deal with socio-economic influencing factors and background. Therefore, our study focused on the trend of MI and ACS incidence, mortality, and MIR among women in Serbia over seventeen years of socioeconomic changes, particularly in relation to HDI and SDI. Our study found a significant decrease in MI incidence by 2017. year for all age groups; the trend of ACS incidence does not change significantly during the follow-up period. The MI mortality trend was decreasing for all age groups, particularly among younger women, until 2019. At the same time, ACS mortality was significantly reduced in younger age groups during the period 2006–2011, i.e., with a declining trend throughout the entire 0–64 years period. There was a significant decline in MIR, both regarding MI and ACS. The average annual per cent change in ACS MIR consistently declines in the age group of women aged 25–64. The decline in MIR may primarily reflect improvements in the quality and completeness of registry data, as well as the health system's performance in diagnosing and managing acute coronary events. According to the findings of previous studies (5), the trend of MI and ACS incidence and mortality was intriguing to analyze in association to the trend of human and socio-economic development, as perceptible through the recorded increasing HDI and SDI trends in our study. Similar to previous studies (33), HDI was found to be inversely associated with MI and ACS incidence, mortality and MIR in our study population. At the same time, analogous to other studies (2), our findings underscore an inverse association between SDI and the incidence, mortality, and MIR of MI and ACS in women.

Although there are studies and analyses of ACS trends in Serbia (23), a classification that categorizes Serbia, due to its high ACS mortality rates, in the group of countries with a very high risk (6) justified our detailed analysis of ACS among women in the context of different socio-economic influencing factors. Coronary atherosclerosis develops earlier and faster in women in Serbia, with an increase in patients under the age of 45, compared with women in EU developed countries and in the USA. As well, women in Serbia suffer from premature MI and ACS premature mortality compared to high-income countries (24). Similar to previous studies, we recognized several aspects of possible association between socioeconomic factors and the trend pattern of coronary health of women: exposure to risk or protective factors (13), gender characteristics (14, 15), preventive examinations, diagnostics and treatment, all in the socio-economic context (10, 18). These factors might be of interest for further analysis on country level, since there is territorial difference in incidence and mortality rates of ACS in Serbia: the highest values of standardized incidence rates of ACS in 2023, according to available data from reports of Institute for Public Health of Serbia, “Dr Milan Jovanovic Batut” and reports from the Serbian Acute Coronary Syndrome Registry, were observed in South district, the Nišava District (242.0/100,000), while the lowest were recorded in North districts, the Srem District (75.3/100,000). The crude mortality rate from ACS in Serbia was 64.2 per 100,000 inhabitants. In 2023, age-standardized mortality rates for ACS were highest in the Kolubara District (95.4/100,000), and lowest in the West Bačka District (13.8/100,000) (34).

Previous studies indicated that growing socioeconomic trend could elevate risks of cardiovascular issues due to urbanization, changed lifestyle, and inadequate behavior regarding risk factors (13, 35). Global burden of 87 risk factors from 2019., indicated that exposure to risk factors could increase, as countries and territories increase SDI, and this could be perceived as a socio-economic development phase (17). Data concerning behavior style in Serbia from 2019 showed that tobacco use was widespread, and heated tobacco products and electronic cigarettes started to be a new challenge (there were 35.1% smokers among women aged 18–64 years in Serbia) (36). There was less physical activity, and eating habits were worse in Serbia than in high-income countries (36).

High MI and ACS mortality rates among women were analyzed globally through studies that emphasized gender differences (5). Influencing factors were related to biology and ACS pathophysiology, as well as atypical symptoms, comorbidities, late access to examinations and medical care, less frequent revascularization, lack of guideline-based therapy at discharge, and generally lower quality of health care coverage for women (15).

Besides risk factors and gender characteristics, other factors may influence ACS outcomes and trends. Education, as a socioeconomic indicator and component of HDI and SDI, rather than wealth, is most consistently associated with CVD outcomes (18), and women with low levels of education are at a higher risk of illness and death compared to educated women (16). It is one of the United Nations Sustainable Development Goals (SDGs), crucial to improving health and wellbeing (37). Given the increasing trends in HDI and SDI, an increase in the level of education among women during this period might be expected, but it was still insufficient to account for the trend in ACS incidence.

Since risk factors for noncommunicable diseases are multidimensional, environmental risks might be considered, as well, especially through possible association with the socio-economic environment. According to the WHO, air pollution and traffic noise are the two major environmental pollutants that affect health, and there is a causal relationship between air pollution and exposure to PM2.5 and cardiovascular morbidity and mortality (38). Some studies considered chronic diseases as “eco diseases” with its environmental and behavioral contributors (39), given the interconnected nature of socioeconomic conditions and environmental risks, that might be further analyzed.

Other contributing factors to the declining trend in ACS MIR may be related to clinical settings and activities that may have led to improvements in health care quality. Publicly owned health institutions comprise a wide network at primary, secondary and tertiary levels and are overseen by the Ministry of Health. Health care at the primary level is provided by state-owned primary care centres, with a well-developed network of outpatient facilities and offices serving one or more municipalities or towns. Secondary and tertiary health services, organized at the regional and national level, are provided by hospitals as the continuation of diagnostics, treatment and rehabilitation initiated at the primary level, or when specialized care is required (40, 41),. The Ministry of Health continuously put efforts in improving the quality of care through numerous multisectoral and sectoral strategies: the network of PCI centres was gradually expanded during the period from 2010 to 2015., 13 hospitals with PCI availability, with a total of 19 catheterization laboratories, were operating in Serbia, and in the following period, the number of these facilities increased (42). Establishment of Acute Coronary Syndrome registry in 2006 was one of the strategic activities as well (23), since the key role of a clinical registry is to stimulate metrics and guarantee quality and improvement of procedures and health care, as well as to initiate improvements in patient outcomes and mortality (43).

Our study examined a period characterized by the transition and continuous investment in improving the quality of the health system (41). Nevertheless, investment in analytical and planned tasks for prevention and health promotion remains a challenge (44), characterized for other ex-socialist countries in Central and Eastern Europe (CEE) (45). Serbia underwent transformational economic growth during the research period, which implies an increase in HDI and SDI during our research period. However, exposure to risk factors remained high and was often overlooked, as in other CEE countries. In the period 2004–2008. there was a change in the financing of outpatient and inpatient healthcare in Serbia, with more funds allocated for outpatient healthcare, increasing from 1.84% of GDP to 2.18% of GDP (46). This trend ensured greater investments in ambulatory diagnostics and treatment at the primary level of health care, in accordance with the EU8 recommendations from the World Bank research, indicating better access, improved patient care on the primary level of care, and therefore, over the years, more accurate records of women patients with ACS (46). As HDI and SDI contain indicators of economic development, it is likely that the inverse trend of ACS mortality and MIR, perceived in our study, might be correlated with a significant increase in the number of coronary care units until 2014, organization of treatment in coronary outpatient units all over Serbia, shortening the emergency medical services' average time, and application of percutaneous coronary intervention (PPCI).

Previous studies pointed out that the economic and social impact resulting of lost years is connected with premature mortality (47). Assessment of YLL in our study revealed a significant decrease in the trend of YLL: 42.6% was lost in the first five years of monitoring, and a fourfold reduction in the last five years of the observed period. It was expected, as ACS MIR had been steadily decreasing in younger age groups. The decreasing trend in YLL follows the increasing trends in HDI and SDI. This might be a result of more effective treatment and better secondary prevention. Despite a declining trend in YLL due to ACS in women, a systematic review of 40 studies for the period 1990–2021. year, based on two commonly used YLL formulas, classified Serbia in a group of middle-income countries with high rates of lost years due to CVD (including Brazil, India, South Africa), contrary to low levels in high-income countries (including Switzerland, Belgium, Spain, Slovenia, the USA, and South Korea) (48). This indicates that premature CVD mortality and ACS mortality remain a major burden for middle-income countries.

## 5 Limitations

There are several limitations: this study is based on secondary data extracted from the ACS registry, which might cause selection and information biases; the ACS registry's quality might be insufficient due to its current conception; the association between the trend of ASC and socioeconomic factors at the population level may not reflect the causal mechanism at the individual level; the impact of environmental factors was not addressed in this study and could be explored in future research; territorial differences in risk factors, population characteristics, and the burden of cardiovascular diseases were not presented and should also be examined in future studies; due to the ecological design and the use of population-level data, it was not possible to adjust for potential confounders such as age distribution, prevalence of cardiovascular risk factors, or access to healthcare. Therefore, the association between socioeconomic indicators and incidence and mortality rates should be interpreted descriptively rather than causally.

## 6 Conclusion

Our findings underscore an inverse association between rising HDI and SDI trends and the incidence, mortality, and MIR of MI and ACS in women. A significant decline in MI incidence and mortality rates, and a decreasing trend in ACS mortality, may be associated with improvements in diagnostics, guideline-directed therapy, and the capacity and quality of coronary care units. Consistently high ACS incidence and mortality rates among women in Serbia might be attributed to complex characteristics of the socioeconomic transformation phases the country was passing through: high exposure to risk factors and weak health promotion and prevention strategies. Consequently, the constant decline in YLL followed, but the number of lost years remains high.

Recognizing the critical importance of women's health, it is essential to prioritize and strengthen cost-effective, multidisciplinary health promotion and prevention strategies targeting women, implemented at all levels of prevention, adapted to diverse local socioeconomic contexts, and aligned with health and other Sustainable Development Goals that address the political, social, economic, and environmental determinants of health and sustainable development, in order to reduce global disparities in cardiovascular outcomes.

## Data Availability

The datasets presented in this study can be found in online repositories. The names of the repository/repositories and accession number(s) can be found below: https://www.batut.org.rs/index.php?content=77 https://www.batut.org.rs/index.php?category_id=9.

## References

[1] Roth GA Mensah GA Johnson CO Addolorato G Ammirati E Baddour LM. Global burden of cardiovascular diseases and risk factors, 1990–2019: update from the GBD 2019 study. J Am Coll Cardiol. (2020) 76(25):2982–3021. 10.1016/j.jacc.2020.11.010 33309175 PMC7755038

[2] Li X Zhao C Liu M Zhao W Pan H Wang D. Sociodemographic index-age differences in the global prevalence of cardiovascular diseases, 1990–2019: a population-based study. Arch Public Health. (2025) 83(1):2. 10.1186/s13690-024-01454-7 39780273 PMC11715713

[3] Sun J Qiao Y Zhao M Magnussen CG Xi B. Global, regional, and national burden of cardiovascular diseases in youths and young adults aged 15–39 years in 204 countries/territories, 1990–2019: a systematic analysis of global burden of disease study 2019. BMC Med. (2023) 21(1):222. 10.1186/s12916-023-02925-4 37365627 PMC10294522

[4] Zhao Y Li J. Global burden of cardiovascular diseases in young adults aged 20–24 years from 1990 to 2021: an analysis of global burden of disease study 2021. CJC Open. (2026). 10.1016/j.cjco.2025.12.016 PMC13386730 42483021

[5] Kunadian V Qiu W Lagerqvist B Johnston N Sinclair H Tan Y. Gender differences in outcomes and predictors of all-cause mortality after percutaneous coronary intervention (data from United Kingdom and Sweden). Am J Cardiol. (2017) 119(2):210–6. 10.1016/j.amjcard.2016.09.052 27816119

[6] Malki N Koupil I Eloranta S Weibull CE Tiikkaja S Ingelsson E. Temporal trends in incidence of myocardial infarction and ischemic stroke by socioeconomic position in Sweden 1987–2010. PLoS One. (2014) 9(8):e105279. 10.1371/journal.pone.0105279 25170919 PMC4149372

[7] World Bank. Serbia Local Infrastructure and Institutional Development Project (P174251): Concept Project Information Document (PID). Washington, DC: World Bank (2021). Available online at: World Bank Document.

[8] Serbia ifPHo. Incidence and Mortality from Acute Coronary Syndrome in Serbia in 2021. Belgrade: Institute of Public Health of Serbia “Dr Milan Jovanović Batut” (2022).

[9] SCORE2 working group and ESC Cardiovascular risk collaboration. SCORE2 Risk prediction algorithms: new models to estimate 10-year risk of cardiovascular disease in Europe. Eur Heart J. (2021) 42(25):2439–54. 10.1093/eurheartj/ehab309 34120177 PMC8248998

[10] Yusuf S Rangarajan S Teo K Islam S Li W Liu L. Cardiovascular risk and events in 17 low-, middle-, and high-income countries. N Engl J Med. (2014) 371(9):818–27. 10.1056/NEJMoa1311890 25162888

[11] Dutta D Mahajan K Verma L Gupta G Sharma M. Gender differences in the management and outcomes of acute coronary syndrome in Indians: a systematic review and meta-analysis. Indian Heart J. (2024) 76(5):333–41. 10.1016/j.ihj.2024.10.002 39389261 PMC11584376

[12] Siabani S Davidson PM Babakhani M Salehi N Rahmani Y Najafi F. Gender-based difference in early mortality among patients with ST-segment elevation myocardial infarction: insights from Kermanshah STEMI Registry. J Cardiovasc Thorac Res. (2020) 12(1):63–8. 10.34172/jcvtr.2020.10 32211140 PMC7080341

[13] Manfrini O Yoon J van der Schaar M Kedev S Vavlukis M Stankovic G. Sex differences in modifiable risk factors and severity of coronary artery disease. J Am Heart Assoc. (2020) 9(19):e017235. 10.1161/JAHA.120.017235 32981423 PMC7792418

[14] Lichtman JH Leifheit EC Safdar B Bao H Krumholz HM Lorenze NP. Sex differences in the presentation and perception of symptoms among young patients with myocardial infarction: evidence from the VIRGO study (variation in recovery: role of gender on outcomes of young AMI patients). Circulation. (2018) 137(8):781–90. 10.1161/CIRCULATIONAHA.117.031650 29459463 PMC5822747

[15] Knox ECL Mateo-Rodríguez I Daponte-Codina A Rosell-Ortiz F Solá-Muñoz S Codina-Rodríguez A. Gender differences in clinical practice regarding coronary heart disease. A systematic review. J Clin Med. (2025) 14(5):1583. (10.3390/jcm14051583) 40095519 PMC11900247

[16] Lee JR Paultre F Mosca L. The association between educational level and risk of cardiovascular disease fatality among women with cardiovascular disease. Womens Health Issues. (2005) 15(2):80–8. 10.1016/j.whi.2004.11.004 15767198

[17] Murray CJL Aravkin AY Zheng P Abbafati C Abbas KM Abbasi-Kangevari M. Global burden of 87 risk factors in 204 countries and territories, 1990–2019: a systematic analysis for the global burden of disease study 2019. Lancet. (2020) 396(10258):1223–49. 10.1016/S0140-6736(20)30752-2 33069327 PMC7566194

[18] Rosengren A Smyth A Rangarajan S Ramasundarahettige C Bangdiwala SI AlHabib KF. Socioeconomic status and risk of cardiovascular disease in 20 low-income, middle-income, and high-income countries: the prospective urban rural epidemiologic (PURE) study. Lancet Glob Health. (2019) 7(6):e748–e60. 10.1016/S2214-109X(19)30045-2 31028013

[19] Simoni AH Valentin JB Kragholm KH Bøggild H Jensen SE Johnsen SP. Temporal trends in socioeconomic disparity in clinical outcomes for patients with acute coronary syndrome. Cardiovasc Revasc Med. (2023) 56:64–72. 10.1016/j.carrev.2023.05.012 37258374

[20] Conceicao P. Human Development Report 2023/24. Breaking the Gridlock: Reimagining Cooperation in a Polarized World (2024).

[21] GBoDC N. Global Burden of Disease Study 2021 (GBD 2021) Socio-Demographic Index (SDI) 1950–2021. Seattle: United States of America: Institute for Health Metrics and Evaluation (IHME) (2024).

[22] Martinez R Soliz P Caixeta R Ordunez P. Reflection on modern methods: years of life lost due to premature mortality-a versatile and comprehensive measure for monitoring non-communicable disease mortality. Int J Epidemiol. (2019) 48(4):1367–76. 10.1093/ije/dyy254 30629192 PMC6693813

[23] Vasić A Vasiljević Z Mickovski-Katalina N Mandić-Rajčević S Soldatović I. Temporal trends in acute coronary syndrome mortality in Serbia in 2005–2019: an age-period-cohort analysis using data from the serbian acute coronary syndrome registry (RAACS). Int J Environ Res Public Health. (2022) 19(21):14457. 10.3390/ijerph192114457 36361340 PMC9659020

[24] Radojković DĐ Damjanović M Apostolović S Milošević J Stanojević D Koraćević G. Acute myocardial infarction in patients under forty-five years of age: what has changed in ten years? Acta Fac med Naiss. (2024) 41(4):294–309. 10.5937/afmnai41-49993

[25] Vučić I. Mortality from acute coronary syndrome on the territory of Serbia and the jablanica district. Glasnik Javnog Zdravlja. (2024) 98(2):143–58. 10.5937/serbjph2402143V

[26] Van de Werf F Ardissino D Betriu A Cokkinos DV Falk E Fox KA. Management of acute myocardial infarction in patients presenting with ST-segment elevation. The task force on the management of acute myocardial infarction of the European Society of Cardiology. Eur Heart J. (2003) 24(1):28–66. 10.1016/S0195-668X(02)00618-8 12559937

[27] Bassand JP Hamm CW Ardissino D Boersma E Budaj A Fernández-Avilés F. Guidelines for the diagnosis and treatment of non-ST-segment elevation acute coronary syndromes. Eur Heart J. (2007) 28(13):1598–660. 10.1093/eurheartj/ehm161 17569677

[28] Ignjatović A Stojanović M Milošević Z Anđelković Apostolović M Filipović T Rančić N. Cancer of unknown primary—incidence, mortality trend, and mortality-to-incidence ratio is associated with human development index in central Serbia, 1999–2018: evidence from the national cancer registry. Eur J Cancer Care (Engl). (2022) 31(1):e13526. 10.1111/ecc.13526 34672038

[29] Souza CDF Oliveira DJ Silva LFD Santos CDD Pereira MC Paiva JPS. Cerebrovascular disease mortality trend in Brazil (1996 to 2015) and association with human development Index and social vulnerability. Arq Bras Cardiol. (2021) 116(1):89–99. 10.36660/abc.20190532 33566971 PMC8159516

[30] Hu QD Zhang Q Chen W Bai XL Liang TB. Human development index is associated with mortality-to-incidence ratios of gastrointestinal cancers. World J Gastroenterol. (2013) 19(32):5261–70. 10.3748/wjg.v19.i32.5261 23983428 PMC3752559

[31] Khazaei Z Goodarzi E Sohrabivafa M Naemi H Mansori K. Association between the incidence and mortality rates for corpus uteri cancer and human development index (HDI): a global ecological study. Obstet Gynecol Sci. (2020) 63(2):141–9. 10.5468/ogs.2020.63.2.141 32206653 PMC7073362

[32] Team RC. R: A Language and Environment for Statistical Computing. Vienna, Austria: R Foundation for Statistical Computing (2016). Available online at: http://www R-project org/ (Accessed on June 12, 2025).

[33] Roy A Roe MT Neely ML Cyr DD Zamoryakhin D Fox KA. Impact of human development Index on the profile and outcomes of patients with acute coronary syndrome. Heart. (2015) 101(4):279–86. 10.1136/heartjnl-2014-306389 25538134 PMC4345920

[34] Batut” IzjzDMJ. Zdravstveno statistički godišnjak Republike Srbije (2025).

[35] Wang H Abbas KM Abbasifard M Abbasi-Kangevari M Abbastabar H Abd-Allah F. Global age-sex-specific fertility, mortality, healthy life expectancy (HALE), and population estimates in 204 countries and territories, 1950–2019: a comprehensive demographic analysis for the global burden of disease study 2019. Lancet. (2020) 396(10258):1160–203. 10.1016/S0140-6736(20)30977-6 33069325 PMC7566045

[36] The Statistical Office of the Republic of Serbia, Serbia EfDoSi. The 2019 Serbian National Health Survey (2021).

[37] United Nations (UN). Women and the Sustainable Development Goals (SDGs). The 2030 Agenda for Sustainable Development (2015) Available online at: https://www.unwomen.org/en/news/in-focus/women-and-the-sdgs (Accessed on June 12, 2025).

[38] Corda MO Charalampous P Haagsma JA Assunção R Martins C. Mortality burden of cardiovascular disease attributable to ambient PM(2.5) exposure in Portugal, 2011 to 2021. BMC Public Health. (2024) 24(1):1188. 10.1186/s12889-024-18572-0 38678185 PMC11055300

[39] Alizadeh G Gholipour K Azami-Aghdash S Dehnavieh R JafarAbadi MA Azmin M. Social, economic, technological, and environmental factors affecting cardiovascular diseases: a systematic review and thematic analysis. Int J Prev Med. (2022) 13:78. 10.4103/ijpvm.IJPVM_105_20 35706860 PMC9188896

[40] European Observatory on Health Systems and Policies, Bjegovic-Mikanovic V Vasic M Vukovic D Jankovic J. Towards equal access to health services in Serbia world health organization regional office for Europe. Eurohealth (Lond). (2020) 26(1):5–8. Available online at: https://iriswhoint/handle/10665/332482 (Accessed on June 12, 2025).

[41] Bjegovic VVM Vukovic D Jankovic J Jovic Vranes A Santric Milicevic M Terzic Supic Z. Serbia: health system review. Health Syst Transit. (2019) 21(3):1–211. 32851979

[42] Bjegovic-Mikanovic V Vasic M Vukovic D Jankovic J Jovic-Vranes A Santric-Milicevic M. Serbia: coronary and structural heart interventions from 2010 to 2015. EuroIntervention. (2017) 13(Z):Z59–63. 10.4244/EIJ-D-16-00829 28504233

[43] Dawson LP Biswas S Lefkovits J Stub D Burchill L Evans SM. Characteristics and quality of national cardiac registries: a systematic review. Circ Cardiovasc Qual Outcomes. (2021) 14(9):e007963. 10.1161/CIRCOUTCOMES.121.007963 34517724 PMC8452241

[44] Stošić S Karanović N. Health care economics in Serbia: current problems and changes. Vojnosanit Pregl. (2014) 71(11):1055–61. 10.2298/VSP120205002S 25536810

[45] Movsisyan NK Vinciguerra M Medina-Inojosa JR Lopez-Jimenez F. Cardiovascular diseases in central and Eastern Europe: a call for more surveillance and evidence-based health promotion. Ann Glob Health. (2020) 86(1):21. 10.5334/aogh.2713 32166066 PMC7059421

[46] Gajic-Stevanovic M Dimitrijevic S Vuksa A Jovanović D. Health Care System and Spending in Serbia from 2004 to 2008. Belgrade: Institute of Public Health of Serbia “Dr Milan Jovanović Batut (2011).

[47] Jakobsen E Green A Oesterlind K Rasmussen TR Iachina M Palshof T. Nationwide quality improvement in lung cancer care: the role of the Danish lung cancer group and registry. J Thorac Oncol. (2013) 8(10):1238–47. 10.1097/JTO.0b013e3182a4070f 24457234

[48] Rodzlan Hasani WS Muhamad NA Hanis TM Maamor NH Wee CX Omar MA. The burden of premature mortality from cardiovascular diseases: a systematic review of years of life lost. PLoS One. (2023) 18(4):e0283879. 10.1371/journal.pone.0283879 37083866 PMC10121009

