# Supplementary material for: Temporal trends in acute coronary syndrome among women and association with socioeconomic factors—evidence from a middle-income country
Source: Front Glob Womens Health. 2026 Jun 2;7:1750182. doi: 10.3389/fgwh.2026.1750182 (PMC13269274; doi:10.3389/fgwh.2026.1750182)
Supplement: Supplementary file 1 [file Table1.docx]

**Suppl. Table 1. Total number of women and deceased women with MI, and ACS**

| **year** | **Incidence** | | | | | | **Mortality** | | | | | |
| --- | --- | --- | --- | --- | --- | --- | --- | --- | --- | --- | --- | --- |
|  | **MI** | | | **ACS** | | | **MI** | | | **ACS** | | |
|  | 25-64 | 0-64 | 0-75+ | 25-64 | 0-64 | 0-75+ | 25-64 | 0-64 | 0-75+ | 25-64 | 0-64 | 0-75+ |
| **2006** | 1854 | 1860 | 6352 | 2632 | 2640 | 8216 | 563 | 564 | 3120 | 572 | 573 | 3190 |
| **2007** | 1666 | 1673 | 6429 | 2584 | 2591 | 8471 | 493 | 495 | 2911 | 501 | 503 | 3014 |
| **2008** | 1775 | 1785 | 6513 | 2572 | 2584 | 8512 | 484 | 485 | 2708 | 499 | 500 | 2853 |
| **2009** | 1950 | 1955 | 7110 | 2695 | 2700 | 9019 | 450 | 452 | 2670 | 466 | 468 | 2835 |
| **2010** | 1896 | 1901 | 6794 | 2610 | 2617 | 8673 | 409 | 414 | 2526 | 429 | 434 | 2663 |
| **2011** | 2026 | 2033 | 6661 | 2811 | 2823 | 8553 | 332 | 332 | 2157 | 345 | 345 | 2270 |
| **2012** | 1770 | 1775 | 6276 | 2509 | 2515 | 8185 | 380 | 380 | 2287 | 391 | 391 | 2387 |
| **2013** | 1548 | 1548 | 5327 | 2216 | 2216 | 6981 | 355 | 355 | 2107 | 365 | 365 | 2167 |
| **2014** | 1795 | 1797 | 6290 | 2433 | 2435 | 8230 | 355 | 355 | 2068 | 363 | 363 | 2194 |
| **2015** | 1763 | 1769 | 6294 | 2831 | 2841 | 9724 | 314 | 314 | 1917 | 316 | 316 | 1962 |
| **2016** | 1595 | 1600 | 5697 | 2337 | 2345 | 7911 | 258 | 258 | 1760 | 262 | 262 | 1814 |
| **2017** | 1384 | 1386 | 5116 | 2272 | 2279 | 7587 | 317 | 317 | 1806 | 322 | 322 | 1867 |
| **2018** | 1733 | 1735 | 6567 | 2315 | 2318 | 8407 | 253 | 254 | 1782 | 261 | 262 | 1884 |
| **2019** | 1632 | 1633 | 6325 | 2523 | 2524 | 8769 | 230 | 231 | 1821 | 236 | 237 | 1886 |
| **2020** | 1818 | 1821 | 6270 | 2494 | 2499 | 7971 | 255 | 255 | 1750 | 260 | 260 | 1821 |
| **2021** | 1496 | 1500 | 5912 | 2414 | 2420 | 8525 | 214 | 214 | 1637 | 218 | 218 | 1696 |
| **2022** | 1503 | 1504 | 5621 | 2124 | 2126 | 7426 | 213 | 213 | 1854 | 219 | 219 | 1948 |
| **Total** | **29204** | **29275** | **105554** | **42372** | **42473** | **141160** | **25877** | **25922** | **91915** | **26388** | **26433** | **95064** |

**Suppl. Table 2. Crude and standardized incidence and mortality rates for MI, NSTEMI, ACS among women in Serbia in 2006-2022.**

| **year** | **Incidence** | | | | | | | | | | | |
| --- | --- | --- | --- | --- | --- | --- | --- | --- | --- | --- | --- | --- |
|  | **MI** | | | | | | **ACS** | | | | | |
|  | **25-64** | | **0-64** | | **0-75+** | | **25-64** | | **0-64** | | **0-75+** | |
|  | CR | ASR-W | CR | ASR-W | CR | ASR-W | CR | ASR-W | CR | ASR-W | CR | ASR-W |
| **2006** | 91.1 | 75.9 | 60.6 | 37.0 | 166.8 | 74.0 | 38.2 | 31.7 | 25.4 | 15.4 | 49.0 | 24.6 |
| **2007** | 81.7 | 67.2 | 54.7 | 32.8 | 169.5 | 71.5 | 126.7 | 103.9 | 84.7 | 50.6 | 223.4 | 98.6 |
| **2008** | 86.8 | 70.4 | 58.6 | 34.5 | 172.5 | 72.6 | 39.0 | 31.4 | 26.2 | 15.3 | 52.9 | 25.5 |
| **2009** | 95.0 | 76.1 | 64.4 | 37.0 | 189.1 | 78.3 | 131.4 | 105.2 | 88.9 | 51.1 | 239.8 | 102.5 |
| **2010** | 92.0 | 71.9 | 62.7 | 35.0 | 181.4 | 74.7 | 126.7 | 99.1 | 86.3 | 48.2 | 231.6 | 97.6 |
| **2011** | 98.2 | 74.8 | 67.3 | 36.6 | 178.7 | 74.4 | 136.2 | 104.5 | 93.5 | 51.1 | 229.4 | 98.7 |
| **2012** | 86.0 | 64.2 | 59.8 | 31.3 | 169.9 | 66.8 | 122.0 | 91.4 | 84.7 | 44.5 | 221.6 | 89.6 |
| **2013** | 75.8 | 56.2 | 52.7 | 27.2 | 144.9 | 56.7 | 108.5 | 80.6 | 75.4 | 39.0 | 189.9 | 77.0 |
| **2014** | 88.7 | 65.1 | 61.8 | 31.6 | 171.9 | 65.2 | 120.2 | 88.8 | 83.7 | 43.0 | 224.9 | 87.1 |
| **2015** | 88.1 | 66.9 | 61.5 | 32.2 | 172.9 | 66.6 | 141.5 | 105.5 | 98.8 | 51.5 | 267.1 | 104.4 |
| **2016** | 80.6 | 61.3 | 56.3 | 29.9 | 157.3 | 60.6 | 118.0 | 89.9 | 82.5 | 43.9 | 218.5 | 86.0 |
| **2017** | 70.7 | 53.0 | 49.3 | 25.7 | 142.1 | 53.2 | 116.1 | 87.2 | 81.1 | 42.5 | 210.7 | 82.8 |
| **2018** | 89.7 | 67.8 | 62.5 | 32.9 | 183.4 | 68.2 | 119.9 | 90.9 | 83.5 | 44.1 | 234.8 | 89.2 |
| **2019** | 85.6 | 65.2 | 59.6 | 31.6 | 177.6 | 65.7 | 132.4 | 101.2 | 92.1 | 49.0 | 246.2 | 95.0 |
| **2020** | 96.7 | 74.5 | 67.3 | 36.2 | 177.2 | 68.3 | 132.6 | 101.8 | 92.3 | 49.5 | 225.2 | 89.2 |
| **2021** | 80.6 | 62.6 | 56.1 | 30.5 | 168.6 | 62.2 | 130.0 | 100.6 | 90.5 | 49.0 | 243.1 | 93.3 |
| **2022** | 84.1 | 63.8 | 58.1 | 30.9 | 164.6 | 60.7 | 118.9 | 90.0 | 82.4 | 43.7 | 217.5 | 82.4 |
| **year** | **Mortality** | | | | | | | | | | | |
|  | **MI** | | | | | | **AKS** | | | | | |
|  | **25-64** | | **0-64** | | **0-75+** | | **25-64** | | **0-64** | | **0-75+** | |
|  | **CR** | **ASR-W** | **CR** | **ASR-W** | **CR** | **ASR-W** | **CR** | **ASR-W** | **CR** | **ASR-W** | **CR** | **ASR-W** |
| **2006** | 27.7 | 23.3 | 18.4 | 11.3 | 81.9 | 32.3 | 28.1 | 23.6 | 18.7 | 11.5 | 83.8 | 32.9 |
| **2007** | 24.2 | 20.2 | 16.2 | 9.9 | 76.8 | 29.2 | 24.6 | 20.6 | 16.4 | 10.0 | 79.5 | 30.1 |
| **2008** | 23.7 | 19.4 | 15.9 | 9.4 | 71.7 | 27.0 | 24.4 | 19.9 | 16.4 | 9.7 | 75.5 | 28.2 |
| **2009** | 21.9 | 17.3 | 14.9 | 8.5 | 71.0 | 25.8 | 22.7 | 17.9 | 15.4 | 8.8 | 75.4 | 27.2 |
| **2010** | 19.8 | 15.0 | 137.0 | 7.5 | 67.4 | 23.9 | 20.8 | 15.7 | 14.3 | 7.8 | 71.1 | 25.1 |
| **2011** | 16.1 | 12.1 | 11.0 | 5.8 | 57.9 | 20.1 | 16.7 | 12.6 | 11.4 | 6.1 | 60.9 | 21.2 |
| **2012** | 18.5 | 13.5 | 12.8 | 6.6 | 61.9 | 20.8 | 19.0 | 14.0 | 13.2 | 6.8 | 64.6 | 21.6 |
| **2013** | 17.4 | 12.4 | 12.1 | 6.0 | 57.3 | 18.8 | 17.9 | 12.8 | 12.4 | 6.2 | 59.0 | 19.4 |
| **2014** | 17.5 | 12.8 | 12.2 | 6.2 | 56.5 | 18.5 | 17.9 | 13.1 | 12.5 | 6.3 | 60.0 | 19.6 |
| **2015** | 15.7 | 11.4 | 10.9 | 5.5 | 52.7 | 16.9 | 15.8 | 11.5 | 11.0 | 5.6 | 53.9 | 17.3 |
| **2016** | 13.0 | 9.3 | 9.1 | 4.5 | 48.6 | 15.3 | 13.2 | 9.5 | 9.2 | 4.6 | 50.1 | 15.7 |
| **2017** | 16.2 | 11.8 | 11.3 | 5.7 | 50.2 | 16.0 | 16.5 | 12.0 | 11.5 | 5.8 | 51.8 | 16.5 |
| **2018** | 13.1 | 9.5 | 9.2 | 4.7 | 49.8 | 15.4 | 13.5 | 9.8 | 9.4 | 4.8 | 52.6 | 16.2 |
| **2019** | 121.0 | 9.0 | 8.4 | 4.4 | 51.1 | 15.3 | 12.4 | 9.2 | 8.6 | 4.5 | 53.0 | 15.8 |
| **2020** | 13.6 | 10.3 | 9.4 | 5.0 | 49.5 | 15.3 | 13.8 | 10.5 | 9.6 | 5.1 | 51.5 | 15.9 |
| **2021** | 11.5 | 8.6 | 8.0 | 4.2 | 46.7 | 14.0 | 11.7 | 8.8 | 8.2 | 4.3 | 48.4 | 14.5 |
| **2022** | 11.9 | 9.0 | 8.3 | 4.3 | 54.3 | 15.8 | 12.3 | 9.2 | 8.5 | 4.5 | 57.0 | 16.6 |

ASR-W -age standardized rate

**Suppl. Table No 3. YLL of ACS among women in Serbia, in the period 2006.-2022.**

| year | YLL | % |
| --- | --- | --- |
| 2006 | 7583.8 | 9.513416 |
| 2007 | 6899.6 | 8.655128 |
| 2008 | 6879.4 | 8.629789 |
| 2009 | 6670.2 | 8.36736 |
| 2010 | 5931.0 | 7.440079 |
| 2011 | 4707.4 | 5.905147 |
| 2012 | 5215.8 | 6.542904 |
| 2013 | 4615.0 | 5.789237 |
| 2014 | 5004.6 | 6.277966 |
| 2015 | 2996.1 | 3.758425 |
| 2016 | 3094.5 | 3.881862 |
| 2017 | 3920.6 | 4.918154 |
| 2018 | 3360.5 | 4.215543 |
| 2019 | 3127.0 | 3.922631 |
| 2020 | 3426.8 | 4.298712 |
| 2021 | 2443.6 | 3.065347 |
| 2022 | 3841.0 | 4.818301 |
| Total | 79716.9 | 100.0 |
